# Overexpression of a novel small auxin-up RNA gene, *OsSAUR11*, enhances rice deep rootedness

**DOI:** 10.1186/s12870-023-04320-w

**Published:** 2023-06-14

**Authors:** Kai Xu, Qiaojun Lou, Di Wang, Tiemei Li, Shoujun Chen, Tianfei Li, Lijun Luo, Liang Chen

**Affiliations:** 1 Shanghai Agrobiological Gene Center, Shanghai, 201106 China; 2 Key Laboratory of Grain Crop Genetic Resources Evaluation and Utilization, Ministry of Agriculture and Rural Affairs, Beijing, China; 3 College of Plant Science & Technology, Huazhong Agricultural University, Wuhan, 430070 China

**Keywords:** Rice, Deep rooting, SAUR, Auxin, Drought resistance

## Abstract

**Background:**

Deep rooting is an important factor affecting rice drought resistance. However, few genes have been identified to control this trait in rice. Previously, we identified several candidate genes by QTL mapping of the ratio of deep rooting and gene expression analysis in rice.

**Results:**

In the present work, we cloned one of these candidate genes, *OsSAUR11*, which encodes a small auxin-up RNA (SAUR) protein. Overexpression of *OsSAUR11* significantly enhanced the ratio of deep rooting of transgenic rice, but knockout of this gene did not significantly affect deep rooting. The expression of *OsSAUR11* in rice root was induced by auxin and drought, and OsSAUR11-GFP was localized both in the plasma membrane and cell nucleus. Through an electrophoretic mobility shift assay and gene expression analysis in transgenic rice, we found that the transcription factor OsbZIP62 can bind to the promoter of *OsSAUR11* and promote its expression. A luciferase complementary test showed that OsSAUR11 interacts with the protein phosphatase OsPP36. Additionally, expression of several auxin synthesis and transport genes (e.g., *OsYUC5* and *OsPIN2*) were down-regulated in *OsSAUR11*-overexpressing rice plants.

**Conclusions:**

This study revealed a novel gene *OsSAUR11* positively regulates deep rooting in rice, which provides an empirical basis for future improvement of rice root architecture and drought resistance.

**Supplementary Information:**

The online version contains supplementary material available at 10.1186/s12870-023-04320-w.

## Introduction

Drought and water shortage are global environment problems that accounts for the great source of crop loss among all natural disasters, and it thus seriously affects the global food security. Rice is one of the main food crops, as it is a dietary staple for more than half of the global population. However, the water consumed in the production of cultivated rice accounts for nearly 70% of irrigation water consumption in agriculture [1]. In China, more than 60% of paddy fields are at risk of drought stress, which seriously affects the production of cultivated rice. Therefore, studying the molecular mechanism of rice drought responses is useful for developing new rice varieties with improved stress resistance and for ultimately reducing the irrigation water consumption and yield loss [2].

Enhancing the water absorption capacity of roots is a critical strategy for rice to avoid damage caused by drought stress [3]. The previous studies on root traits have mainly focused on conventional root growth indicators such as root length, root number, root weight, and root depth [4]. Deep roots are particularly important for drought resistance in rice but have scarcely been studied. When the soil surface is dry, only deep roots can absorb water from the subsoil. Therefore, the number and proportion of deep roots, to a large extent, determine the water absorption capacity of plants under drought stress [5]. Some researchers have used the ratio of deep rooting (RDR) to assess this root characteristic, i.e., the ratio of the number of deep roots to the total number of roots [6, 7].

RDRs can be determined through the standard method referred to as the basket method [6]. In this method, the root system is divided into two parts; the shallow roots are defined as the roots distributed at angles of 0–50 degrees relative to the horizontal plane, and the deep roots are defined as the roots distributed at angles of 50–90 degrees. The first discovered major QTL for deep rooting in rice, which is located on chromosome 9 and named *DRO1*, was mapped using the basket method [7]. Introducing a genomic fragment containing *DRO1* into cultivar IR64 significantly improved its RDR and drought resistance [8]. Another major deep root QTL on chromosome 4, *DRO2*, was mapped using three F2 populations [9]. A major QTL for root angle on chromosome 7, *DRO3*, was found using two lines with extreme root phenotypes, cultivar Kinandang Patong and IR64 [10]. However, the identified genes controlling rice RDR remain very scarce.

Auxin was the first plant hormone to be found, and it plays an important role in plant growth and development [11]. Auxin can regulate the expression of hundreds of genes, and the main early auxin response genes span three gene families: *Aux/IAA*, *GH3*, and *SAUR* [12]. The function of members of the *Aux/IAA* and *GH3* gene families have been extensively studied, whereas research on the *SAUR* family has been scarce. The *SAUR* gene family is the largest auxin response factor family specific to plants. In 1987, the first *SAUR* gene was identified in soybean, as an auxin induced transcript. Subsequently, *SAUR* genes were found widely in various plants. With the development of genome sequencing and bioinformatics, 78 *AtSAUR* genes were found in *Arabidopsis thaliana*, 58 *OsSAUR* genes were found in rice and 99 *SlSAUR* genes were found in tomato [13]. Sequence analysis showed that *SAUR* genes had no introns, and most of *SAUR* genes were clustered in the genome. The relative molecular weight of a SAUR protein is generally small, ranging from 10 kD to 27 kD. There is a conserved downstream element *DST* in the 3 ′ untranslated region of *SAUR*. There are one or more *AuxRE* sequence in the promoter region of *SAUR*, and the N-terminus of most SAUR proteins contains a nuclear localization signal [14]. The redundancy among members of the SAUR family decrease the possibility of obvious phenotypes occurring in the *SAUR* deletion mutants, which greatly increases the difficulty of gene function research [13]. Therefore, there are only a few published studies on the *SAUR* gene family so far. The most published reports on SAUR have examined *Arabidopsis thaliana SAUR* genes, for example, *AtSAUR19* [15, 16], *AtSAUR36* [17], and *AtSAUR41* [18]. *AtSAUR41* was reported to play an important role in root development. *SAUR41*- overexpressing lines showed a significant increase in the length of main roots and more vigorous development of lateral roots [18]. SAUR19, as well as other SAURs, have been shown to interact with type 2 C protein phosphatases, negatively regulate PM H^+^-ATPase activity, and mediate auxin-induced cell expansion [15, 16, 19].

Few SAUR genes have been studied in details in rice so far. It is known that *OsSAUR39* is highly expressed in old leaves and plays a negative role in regulating auxin level and transport. Compared with wild-type (WT) controls, overexpression of *OsSAUR39* can inhibit the growth of stem and root and reduce yield in rice [20]. In addition, *OsSAUR45* was strongly expressed in differentiated taproots and adventitious roots. OsSAUR45 protein is localized in the endoplasmic reticulum. *OsSAUR45-*overexpressing rice plants were shorter than WT plants, with shorter roots, narrower leaves, and a lower seed setting rate, which indicated that *OsSAUR45* negatively regulates plant growth and development [21]. The function of other SAUR genes in rice has not yet been reported.

Previously, the RDR of a rice recombinant inbred line population (IRAT109×Zhenshan 97B) was identified by the basket method, and QTL mapping was conducted in our lab. A total of five major QTLs were detected, among which the QTL within RM6-RM240 on chromosome 2 contributed most to the RDR [22]. Additionally, transcriptome analysis of extreme deep rooting and shallow rooting varieties was conducted, and some differentially expressed genes were found [23]. Candidate genes in this RDR QTL interval were screened and analyzed in combination with differential gene expression analysis. In the present study, we cloned and characterized one of these candidate genes, *OsSAUR11*, from IRAT109, a parent line with a high RDR. It was found that overexpression of *OsSAUR11* can significantly increase RDR and drought resistance of rice plants. OsSAUR11 was found to be located in the cell plasma membrane and nucleus and interacted with protein phosphatase PP36. The present study characterized a novel gene that would be used in rice genetic modification to improve root architecture and drought resistance.

## Results

### Isolation of ***OsSAUR11*** and sequence analysis

**Fig. 1:**
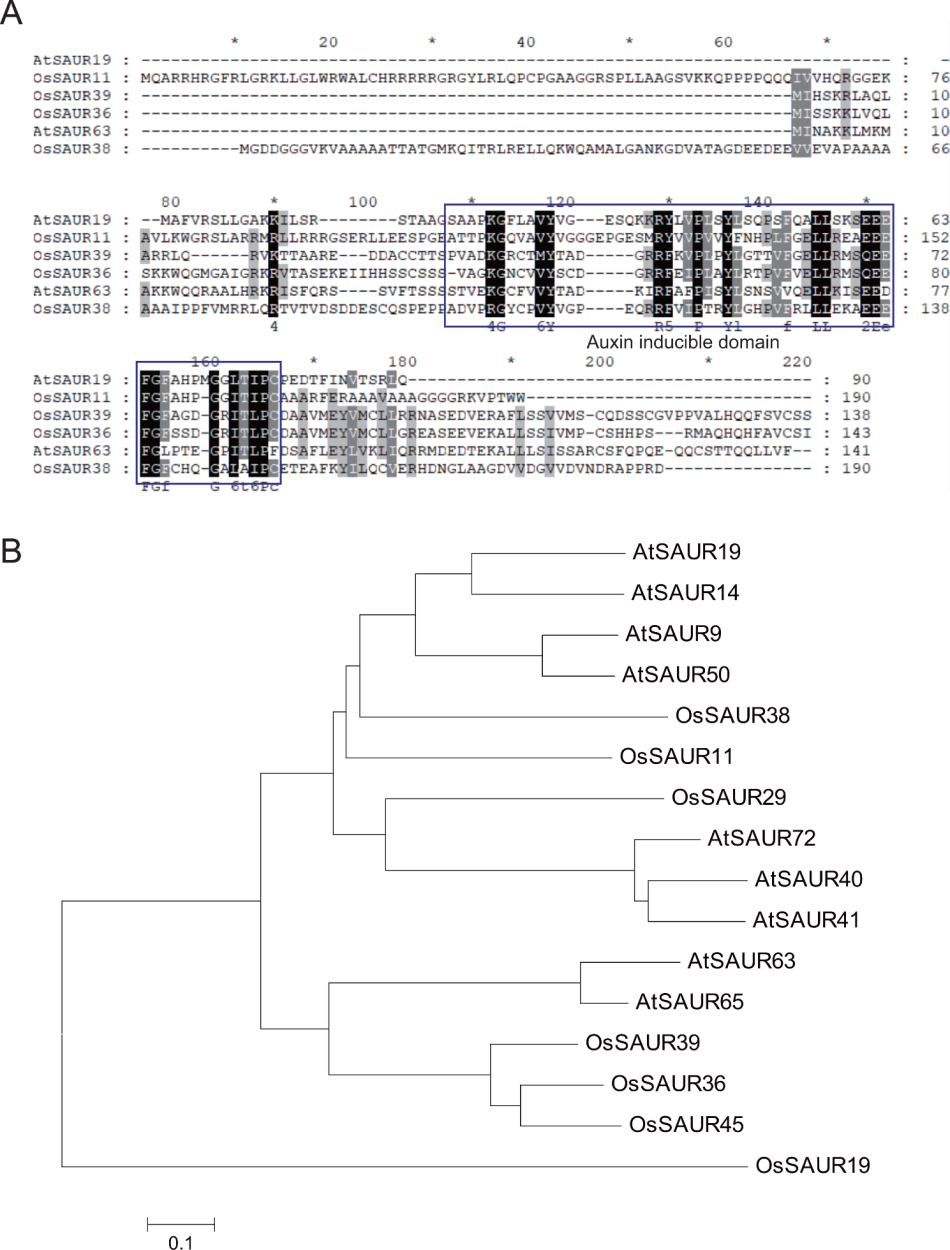
Sequence alignment and phylogenetic analysis of OsSARU11 and other SAUR proteins from *Arabidopsis* and rice. (**A**) Protein sequence alignment of several *Arabidopsis* and rice SAUR subfamily proteins. (**B**) Phylogenetic tree of partial *Arabidopsis* and rice SAUR proteins. The tree was constructed with MEGA5.0. The protein accession numbers in the Arabidopsis Information Resource (www.arabidopsis.org) and Rice Genome Annotation Project (rice.uga.edu) are as follows: AtSAUR9: AT4G36110.1; AtSAUR14: AT4G38840.1; AtSAUR19: AT5G18010.1; AtSAUR40: AT1G79130.1; AtSAUR41: AT1G16510.1; AtSAUR50: AT4G34760.1; AtSAUR65: AT1G29460.2; AtSAUR63: AT1G29440.1; AtSAUR72: AT3G12830.1; OsSAUR11: LOC_Os02g42990.1; OsSAUR19: LOC_Os04g45370.1; OsSAUR29: LOC_Os06g50040.1; OsSAUR36: LOC_Os08g43700.1; OsSAUR38: LOC_Os09g26610.1; OsSAUR39: LOC_Os09g37330.1; OsSAUR45: LOC_Os09g37400.1

Previously, one QTL on chromosome 2 was found to contribute most to rice RDR through QTL mapping analysis. This QTL interval included several candidate genes encoding proteins related to auxin signaling, one of which was *OsSAUR11* (*LOC_Os02g42990*). This gene was cloned from the drought resistant rice IRAT109. There was no difference in the coding region sequence of *OsSAUR11* between Zhenshan 97 and IRAT109, the two parental lines of the recombinant inbred lines used for QTL mapping. The sequence analysis showed that OsSAUR11 encoded a SAUR protein, with an auxin-inducible domain that is conserved with other SAUR proteins in *Arabidopsis* and rice (Fig. 1A). Phylogenetic analysis showed that *OsSAUR11* was similar to its homologs in *Arabidopsis* and rice such as *AtSAUR19* and *OsSAUR38* (Fig. 1B).

### Overexpression and knockout of ***OsSAUR11*** in rice

In order to analyze the function of *OsSAUR11* in rice RDR, we constructed an overexpression (OE) vector of *OsSAUR11* and transformed it into the rice cultivar ‘*Nipponbare’*. Through qPCR analysis of T2 generation transgenic rice, it was found that *OsSAUR11* was overexpressed in most transgenic rice lines compared with WT plants, and three transgenic lines with high expression were selected for the following functional research (Fig. S1). At the same time, we selected a knockout target in the *OsSAUR11* coding region, constructed a CRIPSR/Cas9 gene editing vector, and transformed it into the upland rice line IRAT109. Transgenic lines with mutations in the target were identified through PCR and sequencing of the PCR product. Mutations in *OsSAUR11* were identified in several transgenic lines as single base insertions or deletions and a large deletion (Fig. S2A). Three mutants were chosen for further study.

### Overexpression of ***OsSAUR11*** increased RDR of transgenic rice plants

**Fig. 2:**
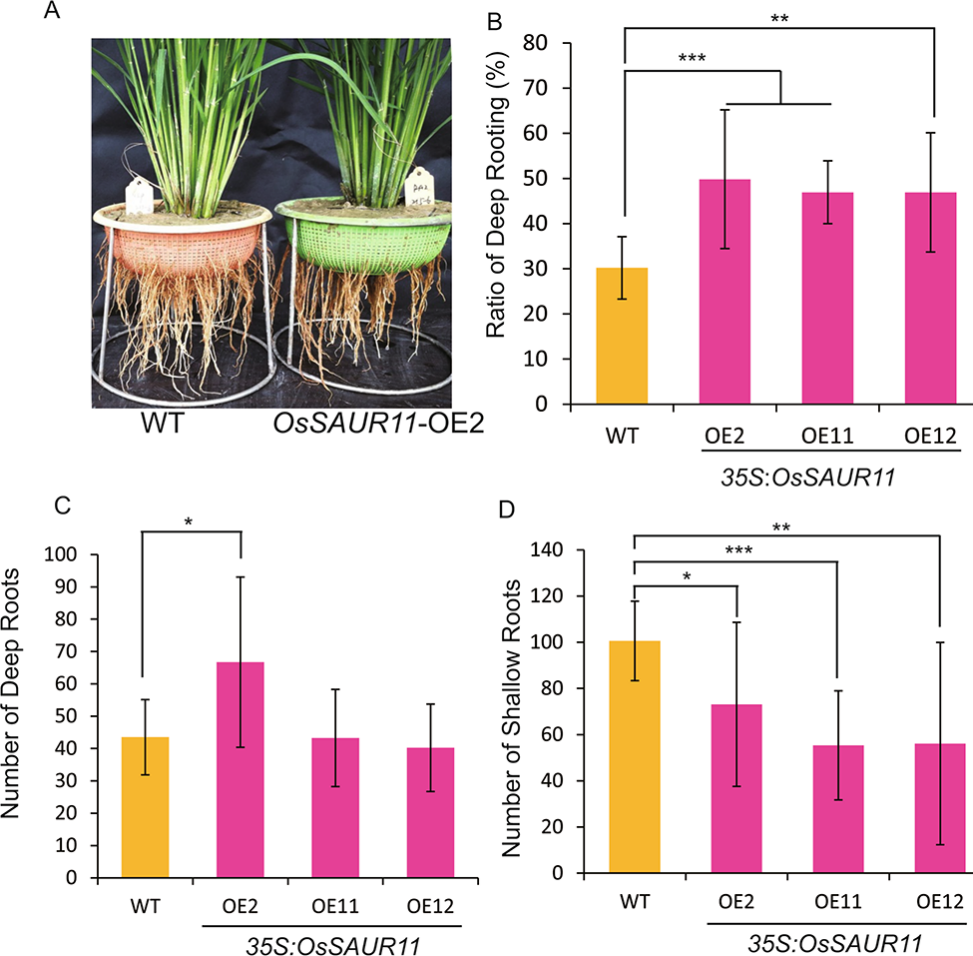
Overexpression of *OsSARU11* increased the ratio of deep rooting of transgenic rice plants. (**A**) Root phenotype of *OsSAUR11*-overexpressing and wild-type (WT) rice plants. (**B**-**D**) Ratio of deep rooting, number of deep roots, and number of shallow roots of transgenic rice plants and WT plants. Error bars indicate the SD of ten biological replicates. * *p* < 0.05, **p < 0.01, and ***p < 0.001, according to Student’s *t*-test

The RDR of *OsSAUR11* transgenic rice plants were further determined. First, the RDR of WT rice plants was about 30.2%, whereas the RDR of the three *OsSAUR11*- over expressing lines ranged from 46.9 to 49.8%, significantly higher than that of WT plants (Fig. 2A and B). The numbers of deep root in *OsSAUR11*-overexpressing rice plants (except for line OE2) were similar to that of WT plants (Fig. 2C), whereas shallow roots were less numerous compared to WT plants (Fig. 2D). Second, there was no significant difference in RDR between *saur11* mutants and that of WT (IRAT109) plants (Fig. S2B). These results showed that overexpression of *OsSAUR11* significantly increased the RDR of transgenic rice, but *OsSAUR11* knockout did not significantly affect rice RDR.

### Root gravitropism and drought resistance of ***OsSAUR11*** transgenic rice plants

**Fig. 3:**
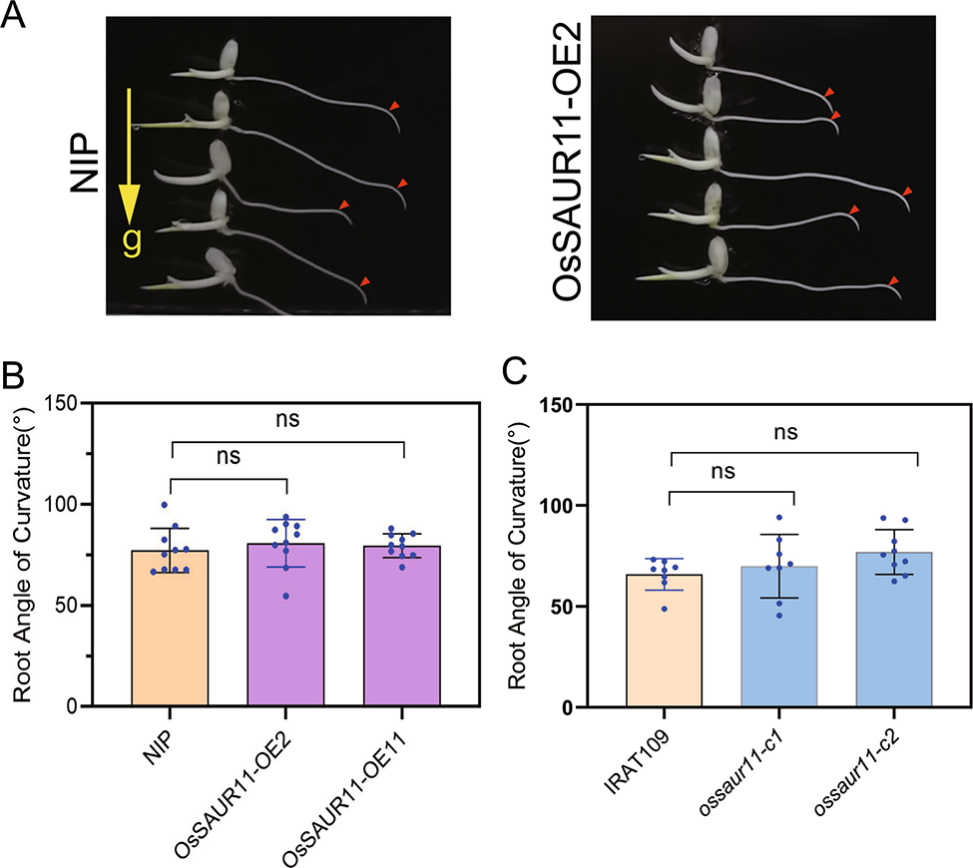
Root gravitropism of *OsSAUR11* transgenic rice plants. (**A**) Root phenotype of *OsSAUR11* OE rice plants after rotated 90° for 3 h. (**B**) and (**C**) Root angle of curvature of *OsSAUR11* OE plants and *ossaur11* mutant plants. Seedlings were grown on MS medium for 4 days and then rotated 90° from the original vertical axis for 3 h. The data represent means ± SE (*n = 10*). Red arrows indicate the positions of root tips at the start of rotation

Gravitropism is one of the major factors affecting the root growth direction. To further analyze the mechanism by which *OsSAUR11* affects the rice RDR, we analyzed the orientation of the primary roots of transgenic rice. After 3 h of rotation, the primary roots bending angle of *OsSAUR11*-overexpressing rice plants was similar to that of WT (i.e., *Nipponbare*) rice plants (Fig. 3A and B). Similarly, there was also no significant difference between the root angle curvature of *saur11* mutants and WT(IRAT109) plants (Fig. 3C). These results showed that the primary root gravity response of transgenic rice was not affected by overexpression or knockout of *OsSAUR11*.

**Fig. 4:**
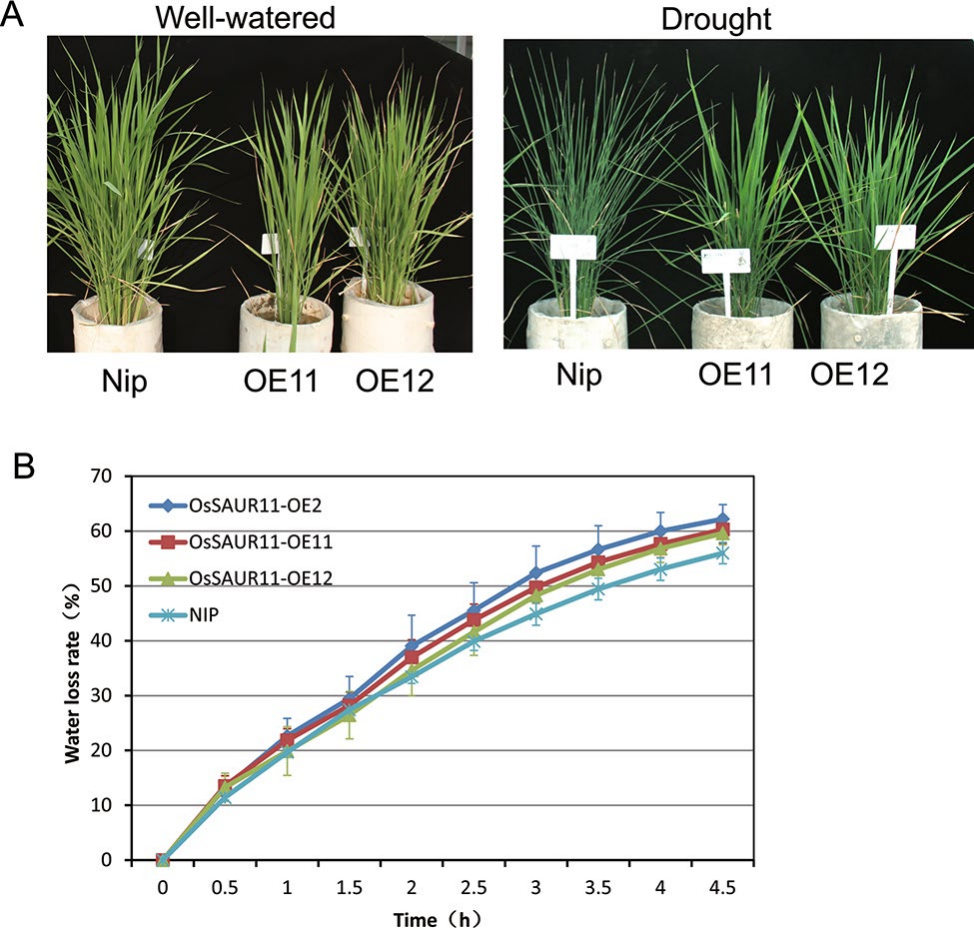
Drought resistance evaluation of *OsSAUR11*-OE rice plants. (**A**) Phenotype of *OsSAUR11*-OE rice plants before and after drought stress treatment. (**B**) Leaf water loss rate of *OsSAUR11*-OE plants. The data represent means ± SE (n = 4)

To evaluate the effect of OsSAUR11 on rice drought resistance, watering of the *OsSAUR11* OE transgenic rice plants were stopped before the young panicle differentiation stage. Leaves of *OsSAUR11*-overexpressing rice plants displayed obvious delayed rolling under drought stress (Fig. 4A). To investigate whether *OsSAUR11* affects leaf water retention, the leaf water loss rates were assessed. The water loss rates of *OsSAUR11*-overexpressing rice plants were slightly higher compared with WT plants but did not reach statistical significance (Fig. 4B), suggesting the improved drought resistance of *OsSAUR11*-overexpressing rice plants could not be mainly attributed to leaf water loss.

### Expression of ***OsSAUR11***

**Fig. 5:**
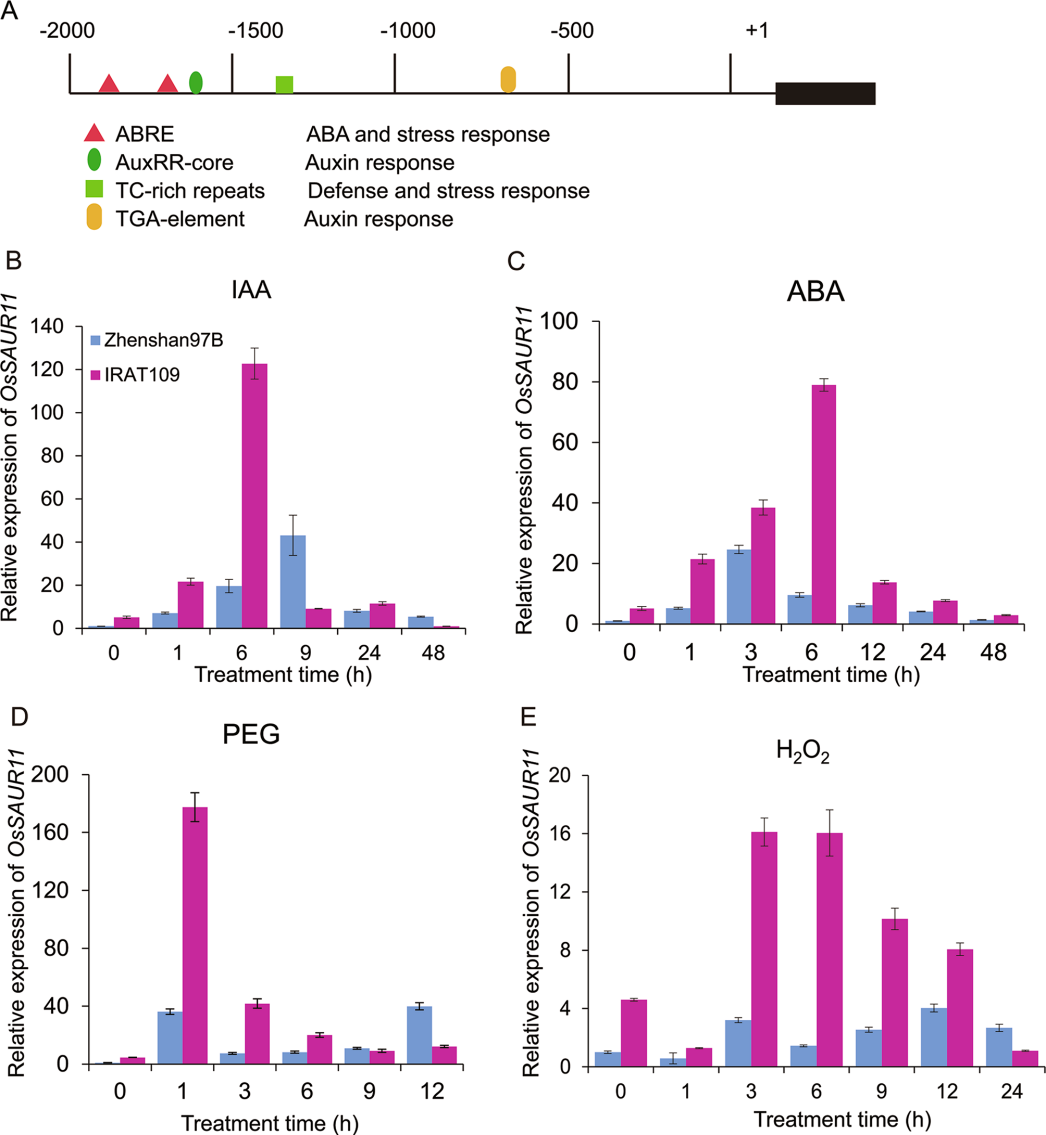
Expression pattern of *OsSAUR11* in roots of IRAT109 and Zhenshan97B rice plants. (**A**) Predicted cis-elements of the *OsSAUR11* promoter. (**B**-**E**) Relative expression of *OsSAUR11* in rice roots under treatments with indole acetic acid (IAA), abscisic acid (ABA), polyethylene glycol (PEG), and H_2_O_2_. Rice seedlings at the four-leaf stage were treated with 0.1 mM IAA, 0.1mM ABA, 15%(*w/v*) PEG6000 and 1%(*v/v*) H_2_O_2_. The whole roots were sampled for expression analysis. The expression levels of *OsSAUR11* were detected by qPCR. Error bars indicate the SE of three replicates

Through bioinformatics analysis, it was found that there were several *cis*-acting elements related to response to auxin, abscisic acid (ABA) and drought stress in the *OsSAUR11* promoter region (Fig. 5A), suggesting that the expression of this gene may be affected by hormones and stress treatments. In order to verify this hypothesis, we analyzed the expression changes of *OsSAUR11* in IRAT109 and Zhenshan97B using qPCR. The expressions of *OsSAUR11* in the roots of the two parental varieties was induced by auxin as well as by polyethylene glycol (PEG) -simulated drought stress, H_2_O_2_, and abscisic acid treatments (Fig. 5B-E). Compared with that in Zhenshan 97B, the expression of *OsSAUR11* in IRAT109 was more obviously induced by these treatments (Fig. 5).

### ***OsSAUR11*** was regulated by the OsbZIP62 transcription factor

**Fig. 6:**
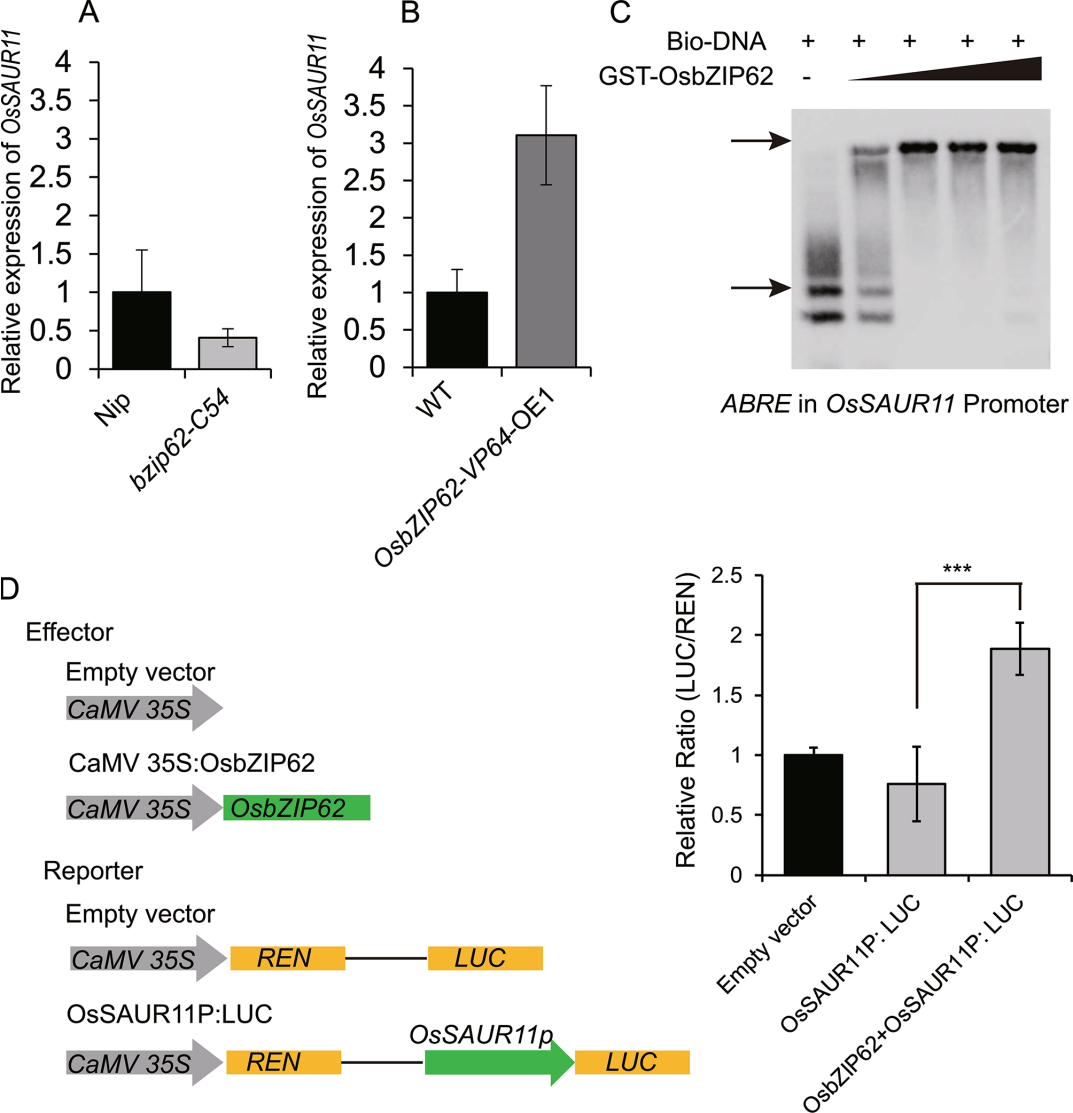
OsbZIP62 may regulate the expression of *OsSAUR11*. (**A**) and (**B**) Relative expression of *OsSAUR11* in *osbzip62* mutant and *OsbZIP62-VP64*-overexpressing rice plants. (**C**) OsbZIP62 protein can bind to the ABRE sequence of *OsSAUR11* promoter in vitro, as indicated by electrophoretic mobility shift assay. The GST-OsbZIP62 protein and biotin-labeled ABRE sequence reacted in vitro and were detected by the biotin reaction. The upper arrow shows the protein DNA binding complex, while the lower arrow indicates the free DNA. (This image has been cropped. For the full-length original blot, see Fig. S4 in Additional file). (**D**) Relative reporter activity in tobacco leaf cells containing *OsSAUR11P* and coexpressed OsbZIP62. The relative LUC activities normalized to the REN activity are shown. Error bars indicate the SD of six biological replicates. ***p < 0.001, according to Student’s *t*-test

The above results showed that expression of *OsSAUR11* was induced by PEG, auxin and ABA treatments. We then investigated whether the expression of *OsSAUR11* was regulated by stress and phytohormone response related transcription factors. In our previous research, OsbZIP62 was identified as a transcription factor involved in ABA and drought responses [24]. Notably, the expression of *OsSAUR11* was significantly higher in *OsbZIP62*-*VP64* transgenic rice plants compared with WT plants, while the expression of *OsSAUR11* in *bzip62* mutants was lower (Fig. 6A and B), suggesting that OsbZIP62 regulates the expression of *OsSAUR11*. Then, we further tested whether OsbZIP62 can directly bind to the promoter of *OsSAUR11*. We expressed and purified the GST-OsbZIP62 fusion protein and detected its DNA binding ability using an electrophoretic mobility shift assay (EMSA) in vitro. When the GST-OsbZIP62 was added into the *OsSAUR11* promoter DNA segment containing the ABRE *cis*-element, a shift band was observed. As the increased OsbZIP62 protein amount, the shift band became darker (Fig. 6C). We further used a transient expression assay to analyze the effect of OsbZIP62 on the expression of the firefly luciferase gene (LUC) driven by the *OsSAUR11* promoter. Co-expression of CaMV35S:*OsbZIP62* with the *OsSAUR11P*: LUC construct led to an significant increase in luciferase activity compared with the *OsSAUR11P*: LUC vector control (Fig. 6D), indicating that OsbZIP62 functions as a transcriptional activator to upregulate the expression of *OsSAUR11*. These results indicated that OsbZIP62 transcription factor can bind to the promoter of *OsSAUR11* and promote its expression.

### Subcellular localization of OsSAUR11 and its interaction with protein phosphatase OsPP36

**Fig. 7:**
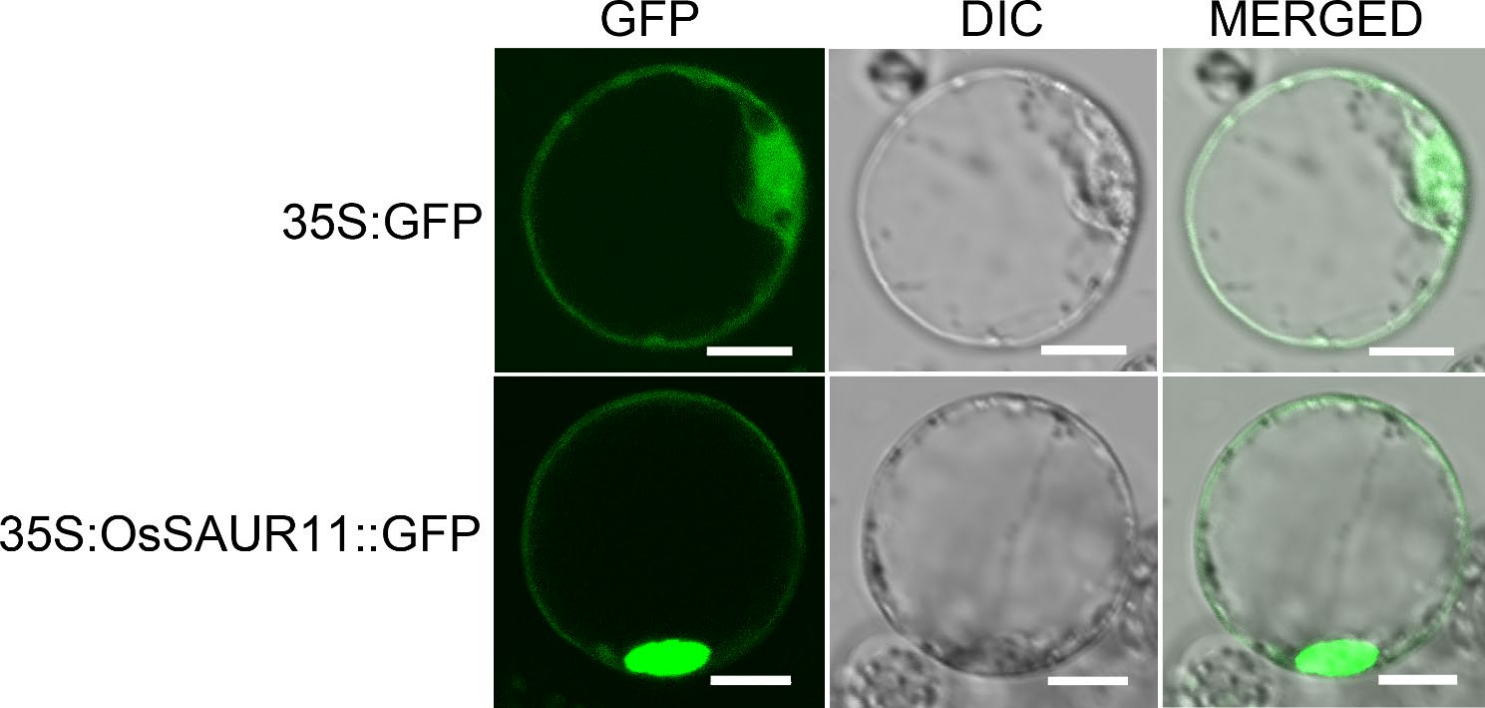
Sub-cellular localization of OsSAUR11. OsSAUR11-GFP fusion proteins were expressed in rice protoplast, and GFP fluorescence was detected by a confocal microscope. DIC, Differential Interference Contrast; MERGE, overlay of GFP and DIC images. Scale bar, 10 μm

To investigate the subcellular localization of OsSAUR11, an OsSAUR11-GFP fusion vector was constructed and firstly introduced to tobacco leaves. The fluorescence signals of OsSAUR11-GFP were detected in the cell membrane and cell nucleus of tobacco epidermal cells (Fig. S3). OsSAUR11-GFP fusion vector was then transformed into rice protoplast. The fluorescence signals of the GFP control were visible throughout the cell, whereas the fluorescence signals of OsSAUR11-GFP were obviously detected in the cell nucleus and cell membrane of rice cells (Fig. 7).

**Fig. 8:**
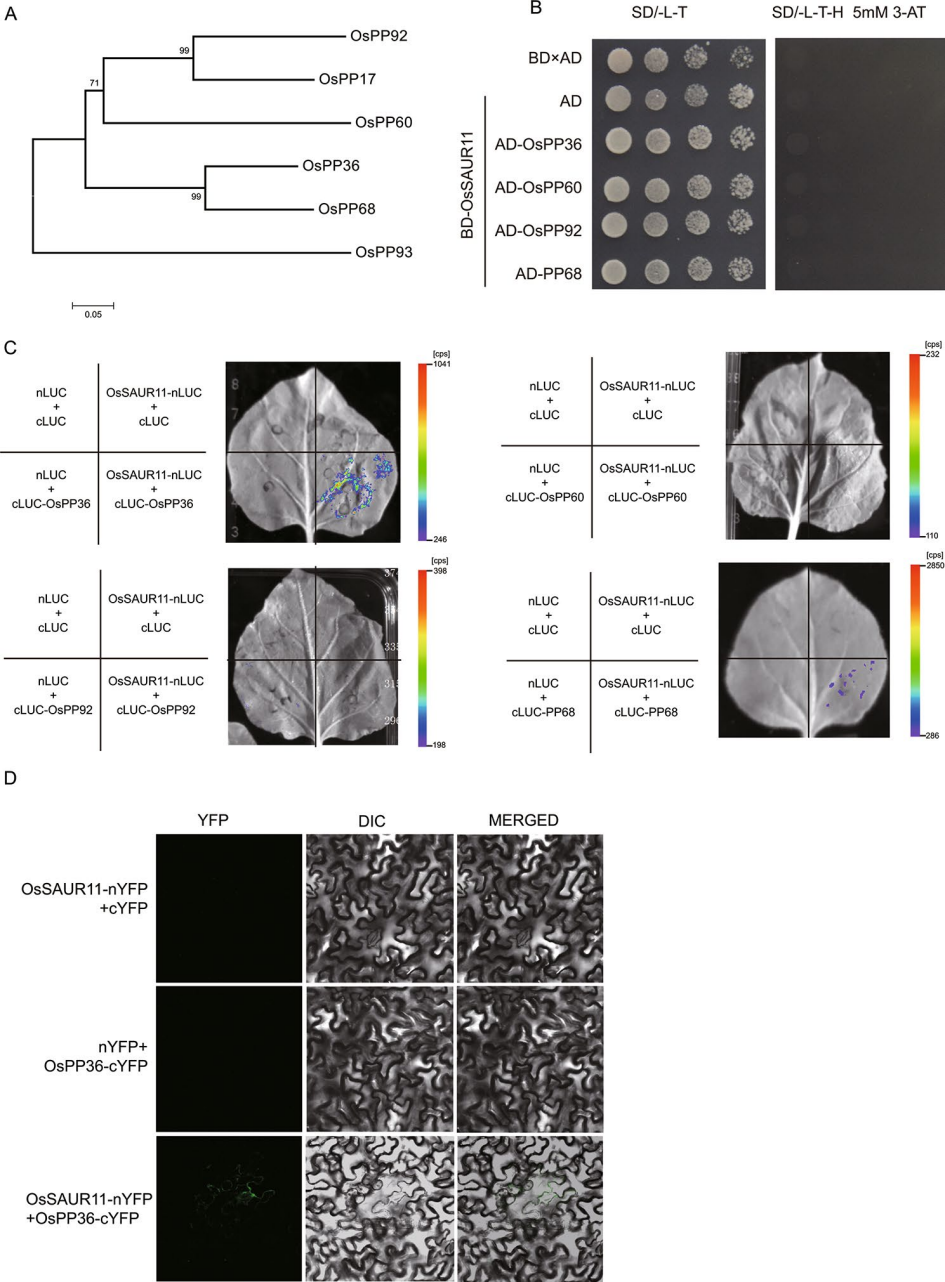
OsSAUR11 interacted with OsPP36. (**A**) Phylogenetic tree of partial rice D-subfamily PP2C proteins. (**B**) Yeast two-hybrid assay of OsSAUR11 and several rice D-subfamily PP2C proteins. (**C**) LUC complementary imaging of OsSAUR11 and several rice D-subfamily PP2C proteins. (**D**) Bimolecular fluorescence complementation analysis between OsSAUR11 and OsPP36 in tobacco leaf tissue

Previous research revealed that AtSAUR19 interacts with the PP2C-D subfamily of type 2 C protein phosphatases (PP2Cs) and affects H^+^-ATPase activity in Arabidopsis. To preliminarily study the molecular mechanism of OsSAUR11, we analyzed whether OsSAUR11 interacts with protein phosphatases. We first cloned several members of the PP2C-D subfamily of PP2Cs in rice, namely OsPP36, OsPP60, OsPP68, and OsPP92 (Fig. 8A), and analyzed the interactions through a yeast two hybrid assay. It was found that there was no obvious interaction between OsSAUR11 and these PP2Cs in yeast (Fig. 8B). We also investigated whether OsSAUR11 interacts with these PP2Cs in vivo through luciferase complementary imaging assay in tobacco leaves. Though OsPP60, OsPP68, and OsPP92 displayed no interaction with OsSAUR11, OsPP36 showed an obvious interaction with OsSAUR11 in tobacco leaf cells (Fig. 8C). Bimolecular fluorescence complementation (BiFC) analysis in tobacco leaves further confirmed that OsSAUR11 can interact with OsPP36 (Fig. 8D).

### OsSAUR11 affects the expression of auxin signaling genes

**Fig. 9:**
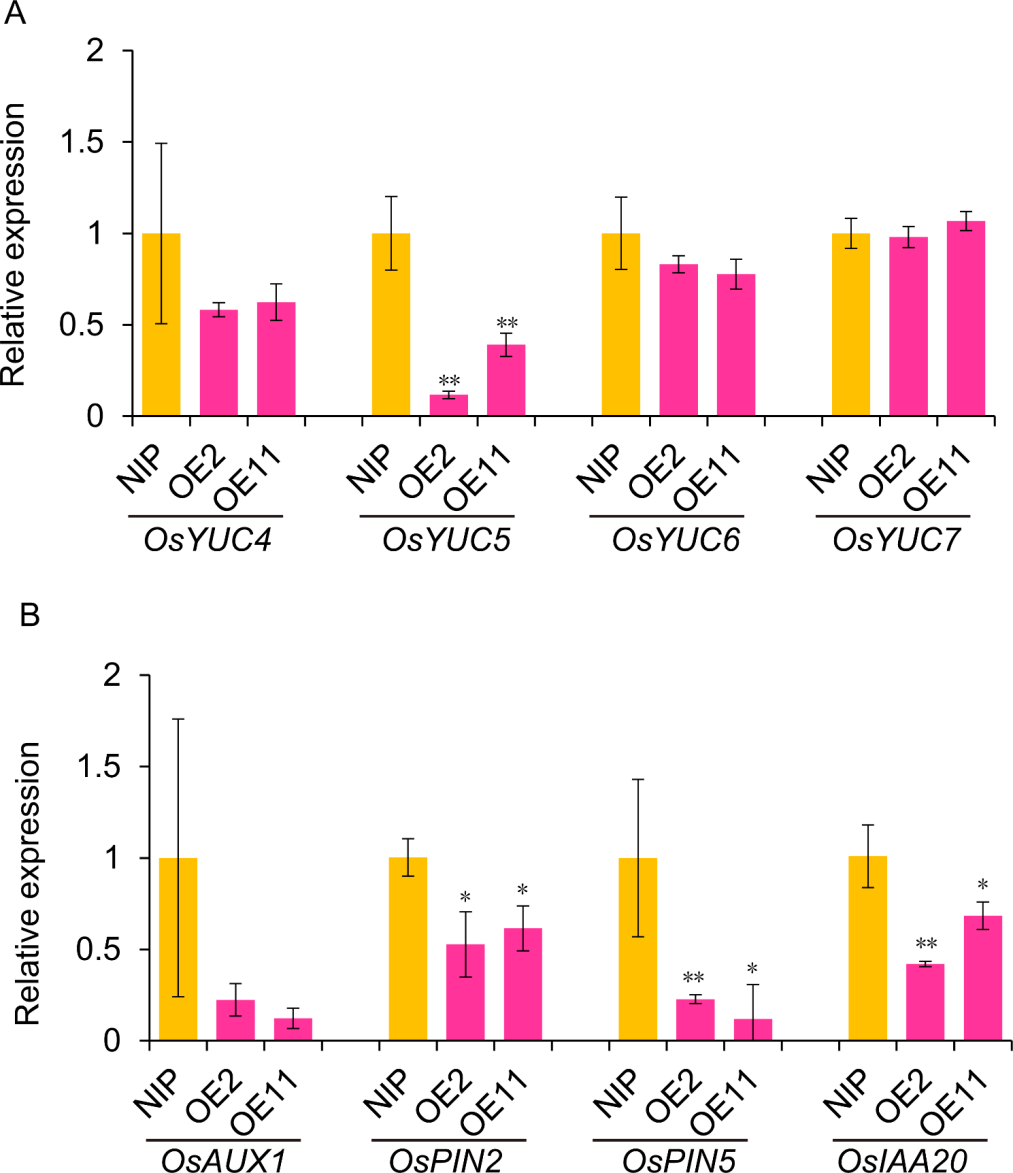
Expression of auxin synthesis and transport genes in roots of *OsSAUR11*-overexpression (OE) transgenic lines. (**A**) The expression of several *YUC* genes in roots of *OsSAUR11*-OE plants. (**B**) The expression of several *AUX* and *PIN* genes in roots of *OsSAUR11*-OE plants. The expression was analyzed through qPCR. NIP, Wild type rice plants; OE2 and OE11, *OsSAUR11*-OE transgenic lines. The data represent means ± SE (*n = 3*). * *p* < 0.05, and **p < 0.01, according to Student’s *t*-test

To further investigate the effect of OsSAUR11 on auxin signaling, we checked the expression of auxin related genes in the roots of *OsSAUR11* OE transgenic rice plants using qPCR. Some auxin synthesis genes (e.g., *OsYUC6* and *OsYUC7*) were expressed similarly in OE and WT plants, whereas the expression of *OsIAA20*, an indicator of auxin signaling, was reduced in *OsSAUR11* OE plants (Fig. 9). Consistently, several genes related to auxin synthesis (i.e., Os*YUC5*) and transport (i.e., Os*AUX4*, *OsPIN2* and *OsPIN5*) were down-regulated in OE plants compared with WT plants (Fig. 9).

## Discussion

### OsSAUR11 is a novel gene that regulates deep rooting in rice

Root architecture plays an important role in plant growth and adaptation to drought, and both nitrogen and phosphorus deficiency. Many genes that control the growth of main roots, lateral roots and root hairs have been identified. However, only a few genes that controlled deep rooting have been discovered. In *Arabidopsis thaliana*, few genes modulating root architecture have been identified. For example, *EXO73* is a newly discovered locus obtained by genetic mapping of *Arabidopsis* root architecture under auxin treatment [25]. For rice, the development of deep roots can be adaptive under drought. The first gene identified by fine mapping and functional identification of RDR QTL was *DRO1*, which was revealed to be one of the main loci regulating deep rooting in rice, playing an important role in the response of rice to drought stress [7, 8]. In this study, we identified another candidate gene in the rice RDR QTL interval and found that overexpression of *OsSAUR11* could significantly increase the rice RDR (Fig. 2) and improve rice drought resistance (Fig. 4A). As overexpression of *OsSAUR11* did not change the leaf water loss rate (Fig. 4B), the enhanced stress resistance could be attributed to the improved RDR. It was also found that knockout of *OsSAUR11* did not affect either the RDR or root gravitropism (Fig.S2 and Fig. 3C). As there are 58 SAUR genes in the rice genome, the lack of a change in phenotype of *ossaur11* mutant may be associated with the functional redundancy of its homologous genes. Knockout of more SAUR genes in a single mutant line would be necessary to characterize their functions in rice.

### ***OsSAUR11*** is involved in crosstalk among auxin, ABA, and drought responses

Auxin induced the rapid expression of three types of genes, namely Aux/IAA, GH3, and SAUR. Many studies have shown that Aux/IAA and GH3 play important roles in auxin signaling, while the function of SAUR-like proteins has been less studied [26]. A few reports in *Arabidopsis* and rice show that SAUR plays an important role in plant growth and development [19]. For example, expression of *SAUR19* is induced by auxin. Functional studies show that *SAUR19* and its homologous genes are involved in auxin induced cell expansion [15, 16]. In the present study, we identified a novel SAUR gene in rice, *OsSAUR11*, whose expression was similarly induced by auxin (Fig. 5). The expression of *OsSAUR11* in IRAT109 (a high RDR line) were higher than that in Zhenshan97(a low RDR line) (Fig. 9), and overexpression of *OsSAUR11* increased the RDR in rice (Fig. 2). These results demonstrated that enhanced expression of *OsSAUR11* contributes to the increased RDR. The expression of *OsSAUR11* was also induced by ABA and PEG treatments (Fig. 5). The promoter contained ABA responsive element (*ABRE*) sequence (Fig. 5A), and OsbZIP62 transcription factor could bind to this element and activate the expression of *OsSAUR11* (Fig. 6). Previously, OsbZIP62 was reported to participate in the response to dehydration and ABA treatment in rice [24]. These results suggest that under drought stress, *OsSAUR11* was induced by OsbZIP62 and enhanced the RDR to cope with stress. It should be noted that the concentration of IAA and ABA used for the treatments in this study is relatively high. As all compounds may have a side effect on the plants, the optimal concentration of phytohormone treatment will be chosen through studying the physiological effect of each concentration in the future research. Nevertheless, these findings suggest that OsSAUR11 may be involved in crosstalk among auxin, ABA and drought response signaling pathway.

### The possible mechanism of RDR regulation by ***OsSAUR11***

The RDR was mainly controlled by the local auxin synthesis, distribution, and response and by the ability of roots to perform re-programming. Gravitropism, hydrotropism, and other tropism may serve as a secondary trait associated with the RDR. The primary root gravitropism of *DRO1* transgenic rice increased significantly, resulting in an increase of the RDR [8]. In the present study, overexpression of *OsSAUR11* was able to increase the RDR mainly by reducing the growth of shallow roots (Fig. 2). Gravitropism analysis showed that overexpression of this gene did not significantly affect the response of the taproot to gravity (Fig. 3). This is in contrast with the *DRO1* regulatory mechanism. In the present study, OsSAUR11 was localized to the cell membrane and cell nucleus (Fig. S3 and Fig. 7), several genes related to auxin synthesis and transport were down-regulated in *OsSAUR11*-OE plants (Fig. 9), suggesting that OsSAUR11 may be involved in the regulation of auxin synthesis and transport. Previous studies have demonstrated that OsSAUR45 and OsSAUR39 were involved in plant growth through negatively regulating auxin synthesis and transport in rice [20, 21]. Similarly, it is possible that OsSAUR11 reduced the growth of shallow roots by inhibition of auxin synthesis and transport. It should be pointed that the gene expression was investigated in roots, while subcellular localization was observed in protoplast and mesophyll cell in this study. As protoplast and mesophyll cell have a different status and different gene expression, it would be more accurate to use root (e.g. using roots of rice plants transformed with *proOsSAUR11*::*OsSAUR11-GFP*) to study subcellular localization in the future research.

The functional study of some SAUR genes in *Arabidopsis* revealed the main mechanism of SAUR family genes, that is, by interacting with PP2C protein phosphatase. They relieve the inhibition of plasma membrane ATPase by PP2Cs, increasing the H^+^ permeability of the plasma membrane, and the acidification of the plasma membrane promotes cell expansion, thus inducing the elongation of hypocotyl and other developmental changes [15, 16]. In this study, OsSAUR11 was found to interact with the PP2C protein phosphatase OsPP36 (Fig. 8C and D), suggesting that OsSAUR11 might participate in a similar signaling pathway. The detailed mechanism should be further studied.

## Conclusions

In this study, we isolated and identified the novel gene *OsSAUR11*, which positively regulates the RDR in rice through participating in the regulation of auxin signaling. This gene may be useful for genetic modification of root architecture and drought resistance in rice.

## Materials and methods

### Cloning of ***OsSAUR11*** and vector construction

The CDS of *OsSAUR11* was amplified using the template obtained by the reverse transcription of IRAT109 RNA. The PCR product was ligated into the pEASY blunt simple vector, and confirmed by PCR and sequencing. The ORF of *OsSAUR11* was inserted into the over-expression vector pCB4004 by the Invitrogen Gateway recombinant reaction (Invitrogen, Carlsbad, CA, USA). The gene knockout vector was constructed using the CRISPR/Cas9 system as described previously [27]. The gene editing site was designed using the CRISPR-design tool available from the Rice Information GateWay (http://rice.hzau.edu.cn/cgi-bin/rice_rs3/CRISPR_rice). The gRNA expression box was amplified through fusion PCR and ligated into the linearized pYLCRISPR/Cas9 vector using recombinant methods. The full-length *OsSAUR11* genomic DNA including the promoter region (approximately 2 kb upstream) was amplified using the genomic DNA of IRAT109 and was then sequenced.

### Plant treatments and gene expression analysis

For gene expression analysis, the seeds of IRAT109 and Zhenshan97 were grown in 96-well plates with culture media. Approximately 21-day-old rice seedlings were separately treated with 15%(*w/v*) PEG6000, 1%(*v/v*) H_2_O_2_, 0.1mM indole-3-acetic acid (IAA), and 0.1mM ABA. The whole roots were sampled at the indicated times for expression analysis. The total RNA was extracted using the TRIzol-A^+^ reagent kit, and cDNA were synthesized using the one-step gDNA removal and cDNA synthesis super mix kit (Transgene). The target gene expression level was detected by qPCR using the TransStart ® Top green qPCR Supermix kit (Transgene) and CFX96 real-time PCR detection system (Bio-Rad). The rice actin gene *OsACT2* (*Os11g0163100*) was used as the reference gene to normalize the target gene expression, which was calculated using the relative quantification method (2^−ΔΔCT^).

### Genetic transformation of rice and molecular identification of transgenic rice

The above overexpression and gene knockout vectors were transformed into Nipponbare and IRAT109 by *Agrobacterium*-mediated rice genetic transformation, respectively. The transgenic rice plants were selected by regeneration on Murashige and Skoog (MS) medium containing hygromycin. For identification of positive transgenic rice plants, DNA was extracted from the leaves of T_0_ generation transgenic plants and PCR was performed using primers for the vector selection marker *HptII*. To measure target gene expression in transgenic rice plants, RNA was extracted, and RT-qPCR was performed, and the expression level of *OsSAUR11* in transgenic rice plants were calculated as described above. For identification of *ossaur11* mutants, the DNA of T_0_ generation knockout transgenic plants was extracted, and PCR was conducted to amplify the gene editing target in the rice genome; the products were then sequenced and aligned with the gene editing target sequence.

### Identification of the RDR, root response to gravity and drought resistance of transgenic rice plants

The seeds of *OsSAUR11*-overexpressing lines were soaked in 50 mg/L hygromycin solution, and the seeds of *ossaur11* mutant and WT plants were soaked directly in water. Uniformly germinated seeds were selected to be transferred into 96-well plates (125 × 85 mm), and cultivated in the Yoshida nutrient solution. Rice seedlings that have grown for 2 weeks were transplanted into the baskets to be planted in the field. After 40 days, each basket was pulled out and cleaned carefully. Then, the number of roots observed at the side and bottom of the basket was counted to calculate RDR.

To investigate the root response to gravity of transgenic rice, the seeds of *OsSAUR11* OE and WT rice were hulled, sterilized, and then germinated on the ½-strength MS medium containing or not containing 50 mg/L hygromycin for 2 days. The germinated rice seedlings were transferred to the ½-strength MS medium in square Petri dishes. After 4 days, the plates were rotated 90 degrees, and digital images were taken every hour. The response angle of the root tip was analyzed using Image J software.

For the drought resistance test, the *OsSAUR11-OE* and WT rice plants were planted in polyvinyl chloride (PVC) pipes filled with soil and the water supply was halted before the young panicle differentiation stage for about 30 days. After severe wilting of WT plants, all the plants were re-watered until they were harvest. The second leaves of rice plants under normal condition were harvest and weighted every half hour, and the water loss rates of detached leaf were calculated.

### Subcellular localization

The *OsSAUR11* gene was recombined into pCAMBIA1300GFP vector to construct the OsSAUR11-GFP fusion expression vector. The construct was introduced into the *Agrobacterium tumefaciens* strain EHA105, and then injected into 3-week-old *Nicotiana benthamiana* leaves to transiently transform the tobacco epidermal cells as described previously [28]. After incubation for 2–3 days, GFP fluorescence in tobacco epidermal cells was detected using an Olympus FV3000 laser confocal microscope (Olympus). The OsSAUR11-GFP vector was then transformed into rice protoplast as described previously [29]. Briefly, ‘Nipponbare’ rice seeds were germinated in the ½-strength MS medium, and then cultured in the dark for 3 days, after which, the buds with a length of more than 1 cm were picked, moved to the rooting tube, and cultured for 10–12 days at 28 ℃. The leaf sheaths of 15 seedlings were cut into 2-mm-wide fragments. The scabbard was placed in 0.6 M mannitol equilibrium solution and then allowed to equilibrate for 10 min. The enzyme solution was added and incubated for 4–5 h at 28 ℃, and 40 rpm. The protoplasts were filtered with a special filter net and suspended in W5 solution, and protoplasts were collected by centrifugation, resuspended with MMG, and kept it in the dark. The GFP or OsSAUR11-GFP plasmids were transformed into protoplasts by the PEG-mediated method. After being cultured at 28 ℃ for 12–16 h, GFP fluorescence was detected using an Olympus FV3000 laser confocal microscope (Olympus).

### Prokaryotic expression of and purification of protein and EMSA assay

The CDS of *OsbZIP62* was ligated into the prokaryotic expression vector pGEX-6P-1, for expression with a GST fusion label, and transformed into *E.coli* DE3. Expression of the fusion protein was induced by 1mM IPTG at 18 ℃, and SDS-PAGE was used to detect the expression. For protein purification, 100 ml of bacteria culture was collected and ultrasonicated. The GST-OsbZIP62 protein was then purified using the Profinia protein purification system (Bio-Rad). The *ABRE* fragment in the *OsSAUR11* promoter was labeled with biotin and then prepared by PAGE purification. EMSA was tested according to the operating instructions of the LightShift Chemiluminescent EMSA kit (Thermo Fisher Scientific). A chemiluminescence imaging system (Bio-Rad) was used to take images.

### Dual-luciferase assays

For the transient transcription activation assays, constructs harboring the firefly LUC gene under the control of *OsSAUR11* promoters in the pGreenII 0800-LUC vector were generated as reporters. The Renila LUC gene under the control of the CaMV 35 S promoter in the pGreenII 0800-LUC vector was used as an internal control. OsbZIP62 under control of CaMV 35 S promoter in pCAMBIA1300 vector was used as the effector. All of these constructs were introduced into the *Agrobacterium tumefaciens* strain EHA105. Different combinations of vectors were injected into 3-week-old *Nicotiana benthamiana* leaves as described above. After incubation for 2–3 days, the LUC activity was quantified with a Dual-Luciferase reporter assay kit (Transgene) using a microplate reader (Biotek). The results are reported as the ratio between the activity of firefly LUC (LUC) and Renilla LUC (REN).

### Yeast two-hybrid assay

The yeast two-hybrid assay was conducted using the Match-marker™ gold yeast two hybrid system (Clontech). *OsSAUR11* was cloned into the GAL4 BD fusion vector pGBKT7-BD to be bait. Genes encoding several PP2C proteins (i.e., OsPP36, OsPP60, OsPP68, and OsPP92) were cloned from rice, and the CDS sequences were recombined into the pGAD-T7 yeast expression vector. The expression vectors pGBKT7-OsSAUR11 and pGAD-T7-PP2Cs were co-transformed into Y2H gold yeast cells by the lithium acetate transformation method. The yeast cells were grown on SD/-Leu/-Trp medium and cultured at 30 ℃ for 48 h. The positive clones were identified by PCR, and then serially diluted 1,10, 100 and 1000-fold, and spotted onto SD/-Leu-Trp medium and SD/-Leu-Trp-His + 5mM 3-AT medium. The yeast growth was observed after 48–96 h.

### LUC complementation assay

For the LUC complementation assay, the coding sequences of OsSAUR11 and each of OsPP36, OsPP60, OsPP68, and OsPP92 were separately cloned into the pCAMBIA1300-nLUC and pCAMBIA1300-cLUC firefly luciferase vectors, respectively. All these constructs were introduced into *A. tumefaciens* strain EHA105 and then co-injected into 3-week-old *N. benthamiana* leaves to transiently transform the tobacco epidermal cells as described above. Plants were then incubated for 2–3 days. Luciferin was sprayed onto leaves, and the fluorescence signal was detected using a plant in vivo imaging system (NightSHADE LB 985, Berthold).

### BiFC assays

The full-length coding sequence of OsSAUR11 was cloned into pC131-nYFP to generate OsSAUR11-nYFP; the full-length coding sequence of OsPP36 was cloned into pC131-cYFP to generate OsPP36-cYFP, resepectively. Both of these constructs were transformed into *Agrobacterium* (strain GV3101), and different mixtures were infiltrated into leaves of *N. benthamiana* as described above. After incubation for 2–3 days, YFP fluorescence of leaves was detected using an Olympus FV3000 laser confocal microscope (Olympus).

## Electronic supplementary material

Below is the link to the electronic supplementary material.

Supplementary Material 1

## Data Availability

The amino acid sequences information of Arabidopsis SAUR proteins used in this study were collected from Arabidopsis Information Resource (www.arabidopsis.org). The genome and protein sequences of rice SAUR and PP2C proteins used in this study are available in Rice Genome Annotation Project (rice.uga.edu). PP2C gene accession numbers: LOC_Os02g46080.1 (OsPP36), LOC_Os03g61690.1 (OsPP60), LOC_Os04g49490.2 (OsPP68), and LOC_Os06g50380.2 (OsPP92).

## Abbreviations

ABA: Abscisic Acid
EMSA: electrophoretic mobility shift assay
IAA: indole-3-acetic acid
LUC: luciferase
OE: overexpression
PEG: polyethylene glycol
PP2C: type 2 C protein phosphatases
PVC: polyvinyl chloride
QTL: quantitative trait locus
RDR: ratio of deep rooting
SAUR: small auxin-up RNA
WT: wild-type

## References

[1] Luo LJ (2010). Breeding for water-saving and drought-resistance rice (WDR) in China. J Exp Bot.

[2] Luo L, Mei H, Yu X, Xia H, Chen L, Liu H (2019). Water-saving and drought-resistance rice: from the concept to practice and theory. Mol Breed.

[3] Farooq M, Wahid A, Lee DJ, Ito O, Siddique KHM (2009). Advances in drought resistance of rice. CRC Crit Rev Plant Sci.

[4] De Dorlodot S, Forster B, Pagès L, Price A, Tuberosa R, Draye X (2007). Root system architecture: opportunities and constraints for genetic improvement of crops. Trends Plant Sci.

[5] O’Toole JC, Bland WL (1987). Genotypic variation in crop plant root systems. Adv Agron.

[6] Kato Y, Abe J, Kamoshita A, Yamagishi J (2006). Genotypic variation in root growth angle in rice (Oryza sativa L.) and its association with deep root development in upland fields with different water regimes. Plant Soil.

[7] Uga Y, Okuno K, Yano M (2011). Dro1, a major QTL involved in deep rooting of rice under upland field conditions. J Exp Bot.

[8] Uga Y, Sugimoto K, Ogawa S, Rane J, Ishitani M, Hara N (2013). Control of root system architecture by DEEPER ROOTING 1 increases rice yield under drought conditions. Nat Genet.

[9] Kitomi Y, Kanno N, Kawai S, Mizubayashi T, Fukuoka S, Uga Y (2015). QTLs underlying natural variation of root growth angle among rice cultivars with the same functional allele of DEEPER ROOTING 1. Rice.

[10] Uddin N, Fukuta Y (2020). A region on chromosome 7 related to differentiation of Rice (Oryza sativa L.) between Lowland and Upland Ecotypes. Front Plant Sci.

[11] Teale WD, Paponov IA, Palme K (2006). Auxin in action: signalling, transport and the control of plant growth and development. Nat Rev Mol Cell Biol.

[12] Hagen G, Guilfoyle T (2002). Auxin-responsive gene expression: genes, promoters and regulatory factors. Plant Mol Biol.

[13] Jain M, Tyagi AK, Khurana JP (2006). Genome-wide analysis, evolutionary expansion, and expression of early auxin-responsive SAUR gene family in rice (Oryza sativa). Genomics.

[14] Newman TC, Ohme-takagi M, Taylor CB, Green PJ, The S, Cell P (2016). DST sequences, highly conserved among plant SAUR genes, target reporter transcripts for Rapid Decay in Tobacco published by : american society of plant biologists (ASPB) linked references are available on JSTOR for this article. Plant Cell.

[15] Spartz AK, Lee SH, Wenger JP, Gonzalez N, Itoh H, Inzé D (2012). The SAUR19 subfamily of SMALL AUXIN UP RNA genes promote cell expansion. Plant J.

[16] Spartz AK, Ren H, Park MY, Grandt KN, Lee SH, Murphy AS (2014). SAUR inhibition of PP2C-D phosphatases activates plasma membrane H+-ATPases to promote cell expansion in Arabidopsis. Plant Cell.

[17] Stamm P, Kumar PP (2013). Auxin and gibberellin responsive Arabidopsis SMALL AUXIN UP RNA36 regulates hypocotyl elongation in the light. Plant Cell Rep.

[18] Kong Y, Zhu Y, Gao C, She W, Lin W, Chen Y (2013). Tissue-specific expression of SMALL AUXIN UP RNA41 differentially regulates cell expansion and root meristem patterning in arabidopsis. Plant Cell Physiol.

[19] Ren H, Gray WM (2015). SAUR Proteins as Effectors of hormonal and environmental signals in Plant Growth. Mol Plant.

[20] Kant S, Bi YM, Zhu T, Rothstein SJ (2009). SAUR39, a small auxin-up RNA gene, acts as a negative regulator of auxin synthesis and transport in rice. Plant Physiol.

[21] Xu YX, Xiao MZ, Liu Y, Fu JL, He Y, Jiang DA (2017). The small auxin-up RNA OsSAUR45 affects auxin synthesis and transport in rice. Plant Mol Biol.

[22] Lou Q, Chen L, Mei H, Wei H, Feng F, Wang P (2015). Quantitative trait locus mapping of deep rooting by linkage and association analysis in rice. J Exp Bot.

[23] Lou Q, Chen L, Mei H, Xu K, Wei H, Feng F (2017). Root transcriptomic analysis revealing the importance of energy metabolism to the development of deep roots in rice (Oryza sativa L). Front Plant Sci.

[24] Yang S, Xu K, Chen S, Li T, Xia H, Chen L (2019). A stress-responsive bZIP transcription factor OsbZIP62 improves drought and oxidative tolerance in rice. BMC Plant Biol.

[25] Ogura T, Goeschl C, Filiault D, Mirea M, Slovak R, Wolhrab B (2019). Root System depth in Arabidopsis is shaped by EXOCYST70A3 via the dynamic modulation of Auxin Transport. Cell.

[26] Wang Y, Zhang T, Wang R, Zhao Y (2018). Recent advances in auxin research in rice and their implications for crop improvement. J Exp Bot.

[27] Ma X, Zhang Q, Zhu Q, Liu W, Chen Y, Qiu R (2015). A robust CRISPR/Cas9 system for Convenient, High-Efficiency Multiplex Genome Editing in Monocot and Dicot plants. Mol Plant.

[28] Liu L, Zhang Y, Tang S, Zhao Q, Zhang Z, Zhang H (2010). An efficient system to detect protein ubiquitination by agroinfiltration in Nicotiana benthamiana. Plant J.

[29] Zhang Y, Su J, Duan S, Ao Y, Dai J, Liu J (2011). A highly efficient rice green tissue protoplast system for transient gene expression and studying light/chloroplast-related processes. Plant Methods.

