# Supplementary material for: Overexpression of a novel small auxin-up RNA gene, OsSAUR11, enhances rice deep rootedness
Source: BMC Plant Biol. 2023 Jun 14;23:319. doi: 10.1186/s12870-023-04320-w (PMC10265830; doi:10.1186/s12870-023-04320-w)
Supplement: Supplementary file 1 — Supplementary Material 1 [file 12870_2023_4320_MOESM1_ESM.docx]

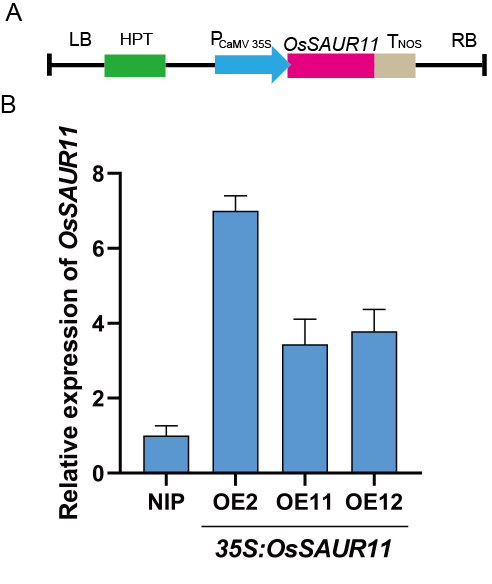


Fig.S1 Identification of *OsSAUR11*-OE transgenic rice plants. (A) The diagram of overexpression vector. (B) Relative expression of *OsSAUR11* in overexpression lines. The expression was detected by qPCR. The data represent means ± SE (n=3).


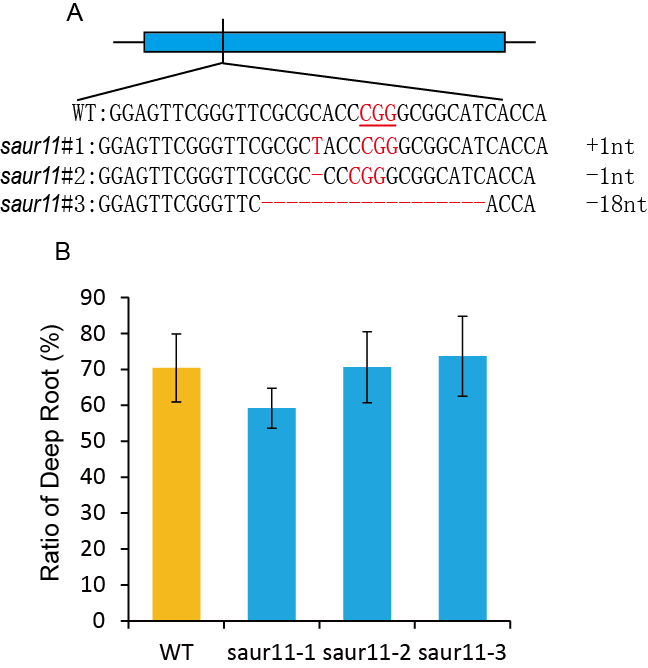


Fig.S2 Knockout of *OsSAUR11* did not affect the ratio of deep root. (A) Sequence in the gene knockout target of *ossaur11* mutants. (B) Ratio of deep root of *ossaur11* mutant. The data represent means ± SE (n=8). WT: wild type (IRAT109).


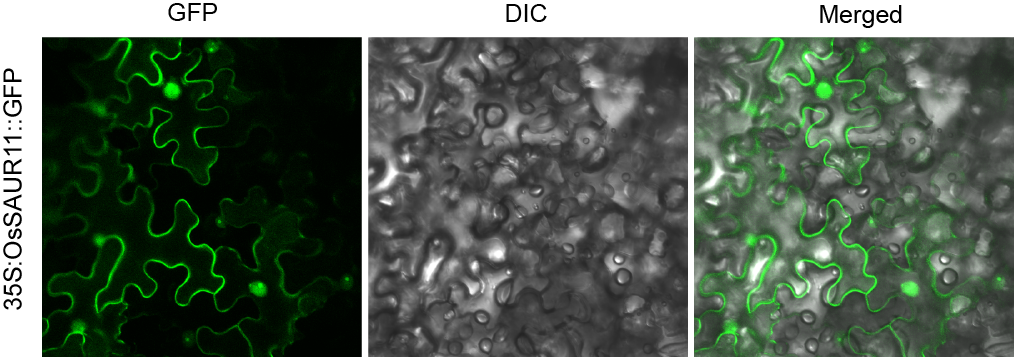


Fig. S3 Subcellular localization of OsSAUR11-GFP. OsSAUR11-GFP fusion proteins were expressed in tobacco leaf epidermal cells, and GFP fluorescence was detected using a confocal microscope. DIC, Differential Interference Contrast; Merged, overlay of GFP and DIC images.


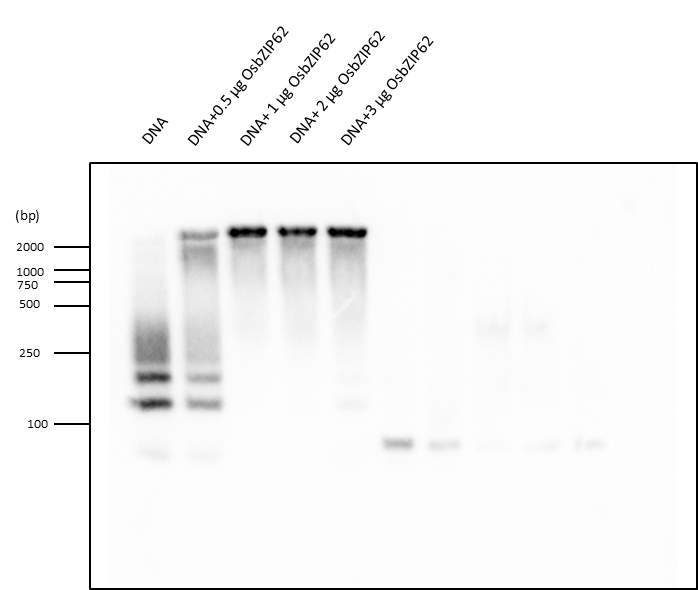


Fig.S4 The original image of Figure 6D. (The black box in the image indicates the boundary between the gels and rad box indicates the region used in figure 6D)
